# Women’s Attitudes Toward Wife-Beating and Intimate Partner Violence (IPV) Among Tanzanian Women

**DOI:** 10.3390/bs16050725

**Published:** 2026-05-08

**Authors:** Nasser B. Ebrahim

**Affiliations:** Department of Public Health, Keimyung University, Daegu 42601, Republic of Korea; nasser.ebrahim@kmu.ac.kr

**Keywords:** social norms, intimate partner violence, wife-beating, Tanzania, Africa

## Abstract

Globally, intimate partner violence (IPV) is a public health concern and the most prevalent type of violence against women. Social norms that condone violence have been strongly associated with intimate partner violence, making them relevant to women’s wellbeing. The identification of social norms unique to a population is necessary for interventions based on shifting social norms. Thus, the current research is aimed at examining the association between women’s attitudes toward wife-beating (a social norm) and women’s experiences of partner’s controlling behavior, physical violence, and emotional and sexual abuse in Tanzania from national representative data. The cross-sectional data used for the study were from Tanzanian women (n = 3033) aged 15–49 years who have ever been married or in a relationship and responded to the domestic violence questionnaire. Nearly, 60% of women reported that their most recent partner had engaged in controlling behavior, 27.5% had experienced physical violence, while 23% and 9.1% had experienced emotional and sexual violence, respectively. The multivariate analysis revealed that IPV was more common among women who had a positive attitude toward wife-beating. In addition to other behavioral and structural interventions, changing societal norms that support violence against women may be helpful to prevent IPV among Tanzanian women.

## 1. Introduction

Intimate partner violence (IPV) refers to violence against women that results in physical, sexual, or psychological harm, and includes controlling behaviors perpetrated by a current or former intimate partner or husband (World Health Organization, 2021, 2024). Globally, in 2021, 26% of women reported experiencing physical and/or sexual violence in their lifetime (World Health Organization, n.d.). IPV disproportionately affects women in sub-Saharan Africa, where lifetime prevalence rates reach up to 44% (Sardinha et al., 2022). In Tanzania, 39% of ever-partnered women have reported experiencing IPV (Ministry of Health et al., 2022). The consequences of IPV include physical injury, death, sexual and mental health problems, and adverse outcomes for children (World Health Organization, 2012). In Tanzania, 41% of women who experienced IPV reported injuries ranging from minor cuts and bruises to severe trauma such as fractures and deep wounds (Ministry of Health et al., 2022).

Achieving gender equality and empowering women and girls, as outlined in Sustainable Development Goal (SDG) 5, requires eliminating violence against women (United Nations, 2015). Risk factors for IPV operate at individual, relational, community, and societal levels (World Health Organization, n.d.). Among these, social norms that condone violence are strongly associated with IPV and considered among the key factors of IPV perpetration (Boyce et al., 2023; Shakya et al., 2022; World Health Organization & London School of Hygiene and Tropical Medicine, 2010). The social norms theory or perspective refers to shared expectations about acceptable behavior within a group (UNICEF, 2021) that can have immense impact on human behavior. Previous work (Cialdini et al., 1991; UNICEF, 2021) identifies three types of norms: descriptive (beliefs about what others do), injunctive norms (beliefs about what others approve of), and personal norms (guides personal behavior through own perception about the appropriateness of the behavior in question). The social norms theory is, however, used in conjunction with hegemonic masculinity and feminist theories to understand intimate partner violence. According to R. Connell (1987) and later refined by R. W. Connell and Messerschmidt (2005), hegemonic masculinity refers to dominant ideals of manhood characterized by emotional restraint, aggression, competitiveness, and the pursuit of authority. These ideals are often sustained through patriarchal social systems that legitimize men’s power over women and subordinate other men (Jewkes & Morrell, 2012). Feminist theory views intimate partner violence as a consequence of women living in societies where violence and aggression are considered acceptable or tolerated, and where such behaviors are used to control and dominate women, reflecting socially sanctioned power imbalances (Amaral, 2011; Krishnan et al., 2012; Rakovec-Felser, 2014). Addressing norms that justify violence—such as the acceptance of wife-beating—can significantly reduce gender-based violence (Gage, 2005; Jewkes, 2002; Johnson & Das, 2009; Clark et al., 2018). Social norms also shape health behaviors by influencing perceptions of others’ actions and attitudes (Berkowitz et al., 2005; Prentice & Miller, 1996). Interventions that correct misperceptions of norms have shown promise in reducing risky behaviors, including IPV (World Health Organization, 2009; Clark et al., 2024).

In Tanzania, patriarchal social structures reinforce male dominance and contribute to norms that perpetuate IPV (Dillip et al., 2018; Saunders et al., 2023). These norms are often internalized by both men and women (McKinley et al., 2021). For instance, 32% of men and 48% of women in Tanzania justify wife-beating, the physical assault of the wife by a husband or intimate partner, under certain circumstances (Ministry of Health et al., 2022). Addressing such norms is therefore important for improving women’s health and wellbeing. Thus, the current study is aimed at understanding the association between women’s attitudes toward wife-beating and their experiences of IPV using nationally representative data from Tanzania.

## 2. Methods

### 2.1. Study Design

For the current study, data from the 2022 Tanzania Demographic and Health Survey and Malaria Indicator Survey (TDHS-MIS) were used. To collect the data, a two-stage stratified sampling design was employed. First, 629 clusters stratified by rural and urban areas were selected. In the second stage, 26 households were selected from each cluster. The analytic sample included 3033 ever-partnered women (women who were ever-married or had romantic partner) aged 15–49 years. Detailed sampling procedures are described elsewhere (Ministry of Health et al., 2022). Survey protocols were approved by the Medical Research Council of Tanzania and the Zanzibar Health Research Institute, and reviewed by ICF International (Ministry of Health et al., 2022).

### 2.2. Measurements

IPV was assessed through four domains: controlling behavior, emotional abuse, physical violence, and sexual violence. The measurement scale used for measuring IPV variables was the modified version of the Conflict Tactics Scale (CTS) (Kishor, 2005). When the respondent acknowledges that any of the specific acts in the scale happened, then the person would be considered to have experienced violence (Kishor, 2005). They were measured from women’s responses to the relevant IPV domains to measure different dimensions of intimate partner violence (see Table 1). The primary independent variable was women’s attitudes toward wife-beating, which measured women’s acceptance of physical assault or violent abuse by a husband or romantic partner. It was measured based on women’s responses to questions asking whether wife-beating is justified under the following scenarios: if a woman goes out without informing the husband, if she neglects her children, if she argues with the husband, if refuses sex, and if she burns the food. Responses were coded as “yes” or “no.” These questions were derived from the Demographic and Health Survey (DHS) to assess women’s acceptance of justification for intimate partner violence. Because responses were recorded as “yes” or “no, ” a nominal variable was constructed to examine differences in violence acceptance norms between groups. The use of a nominal variable was appropriate, as responses inherently classified women into those who accept and those who do not accept wife-beating. Moreover, this approach facilitates easier interpretation of findings for end users compared to relying solely on regression coefficients. Accordingly, women endorsing three or more scenarios were classified as having favorable or positive attitudes toward wife-beating, reflecting women’s acceptance or tolerance of violence, while others were classified as having unfavorable attitudes toward wife-beating.

### 2.3. Data Analysis

Data were analyzed using SPSS version 29 with the complex samples module. Sampling weights, clustering, and stratification were accounted for. Descriptive statistics were used to estimate IPV prevalence. Multivariable logistic regression models were used to assess associations between attitudes and IPV outcomes, after adjusting for covariates including age, residence, region, wealth, education, employment, alcohol use, and decision-making autonomy. Statistical significance was set at *p* < 0.05.

## 3. Results

Most participants (68.6%) resided in rural areas, and 37.3% were from poor households. Approximately 19.7% had no formal education. Among husbands/partners, 13.5% had no formal education, and 90.2% were employed in the past year. About 21.6% of husbands/partners consumed alcohol. Nearly 60% of women reported experiencing controlling behavior, 27.5% reported physical violence, 23% emotional abuse, and 9.1% sexual violence. Additionally, 29.9% of women held favorable attitudes toward wife-beating (Table 2). Multivariable analyses showed that favorable attitudes toward wife-beating were significantly associated with all IPV outcomes. Women with favorable attitudes had higher odds of experiencing controlling behavior (OR = 2.04, 95% CI [1.63, 2.56]), emotional abuse (OR = 1.88, 95% CI [1.45, 2.39]), physical violence (OR = 1.98, 95% CI [1.55, 2.53]), and sexual violence (OR = 2.12, 95% CI [1.47, 3.05]) (Table 3). Husbands’/partners’ alcohol consumption was consistently associated with increased IPV risk across all outcomes (Table 4).

## 4. Discussion

Nearly 60% of women in the study reported that their most recent husband or partner had engaged in at least one controlling behavior. Approximately 28% of women had experienced physical violence, while 23% and 9.1% had experienced emotional and sexual abuse, respectively. These findings are consistent with previous reports. For example, the Ministry of Health et al. (2022) reported that 61% of women experienced one controlling behavior and 27% experienced physical violence, which was similar to other countries in sub-Saharan Africa (Mossie et al., 2023). As substantial proportions of women in Tanzania continue to experience intimate partner violence, it poses a challenge to achieving Sustainable Development Goal (SDG) 5.2, which aims to eliminate violence against women by 2030 (United Nations, 2015). Addressing this issue requires multifaceted efforts to promote gender equality, including school-based, community-based, media, and behavioral interventions (World Health Organization, 2009). Women who held favorable attitudes toward wife-beating were more likely to experience controlling behavior, physical violence, emotional abuse, and sexual violence compared with those who held unfavorable attitudes. This significant association between women’s acceptance of violence and IPV may reflect the influence of patriarchal social norms that shape behavior and perpetuate violence (Carter, 2015). Sociocultural and religious factors rooted in patriarchy contribute to traditional constructions of masculinity that reinforce, normalize, and tolerate violence against women (Carter, 2015; Sikweyiya et al., 2020).

One approach to preventing gender-based violence is to transform social and cultural norms—defined as implicit rules or expectations governing behavior within a group—that enable violent behavior (UNICEF, 2021; World Health Organization, 2009). While some norms discourage violence, others may promote or justify it (World Health Organization, 2009). When violence is accepted as a means of conflict resolution or as part of child-rearing practices, the risk of interpersonal violence increases (World Health Organization, 2002). For example, in Tanzania, nearly 30% of women report favorable attitudes toward wife-beating, while corporal punishment remains a legally accepted child-rearing practice (End Corporal Punishment, 2025). Furthermore, patriarchal societies, including those in Tanzania, have social systems that strongly privilege men and reinforce gender inequalities through rigid norms (Luoga et al., 2025; Saunders et al., 2023). Women’s vulnerability to intimate partner violence may also be linked to their lack of resources, resulting from structural and systemic factors that place women in subordinate and disadvantageous positions, as well as cultural and societal norms that reinforce male dominance (Mshweshwe, 2020; Sikweyiya et al., 2020). Such norms can collectively sustain an environment in which violence against women persists. Interventions aimed at changing social norms have been shown to reduce violence by shifting beliefs that support such behaviors (World Health Organization, 2009; Clark et al., 2024). Mass media campaigns, combined with information on legal consequences, may help shift societal attitudes and contribute to the prevention of IPV (World Health Organization, 2009; Clark et al., 2024).

Among the sociodemographic, environmental, and behavioral factors examined, men’s alcohol consumption was consistently associated with all IPV outcomes. Women whose husbands or partners consumed alcohol were more likely to report controlling behavior, physical violence, emotional abuse, and sexual violence compared with those whose partners did not consume alcohol. This finding is consistent with previous research conducted in Tanzania (Shubina et al., 2023) and elsewhere (Caetano et al., 2017; Leonard & Quigley, 2017). Alcohol use may increase both the frequency and severity of IPV by impairing cognitive and physical functioning and reducing self-control (U.S. Department of Justice, n.d.). In addition, excessive alcohol consumption can exacerbate financial strain, infidelity, and other household stressors. Societal beliefs that associate alcohol use with aggression may further increase the risk of violence (U.S. Department of Justice, n.d.). Given the high prevalence of alcohol use among men in Tanzania and its strong association with IPV, implementing behavioral and policy interventions to reduce hazardous alcohol consumption may be an effective strategy for preventing violence against women. The findings also indicate that women from the Western, Northern, Central, Southern Highlands, and Southwest Highlands zones were more likely to experience emotional abuse than those from the Eastern zone. The Eastern zone, which includes coastal regions and Dar es Salaam—Tanzania’s largest commercial city—may have greater access to resources such as gender equality programs, social support services, and legal assistance.

## 5. Conclusions

A major obstacle to reaching the Sustainable Development Goal of ending violence against women by 2030 is the fact that a sizable percentage of Tanzanian women still endure intimate partner violence (IPV). Women who have a positive attitude toward wife-beating are much more likely to experience IPV. Addressing intimate partner violence (IPV) requires transforming harmful social norms alongside implementing behavioral and structural interventions. Community-based interventions that actively engage members of society and foster meaningful dialog around harmful masculinity norms are essential. In addition, sustained awareness campaigns are needed to challenge and change inequitable gender norms. Also, educational and religious institutions could play a critical role in shaping the attitudes and behavior of future generations. However, because the study is based on cross-sectional data, further research is needed to confirm the results.

## Figures and Tables

**Table 1:** IPV domains used to assess IPV.

IPV Types	Items	Participants’ Response Options	Categories
Controlling behavior	Husband/partner jealous if respondent talks with other men. Husband/partner accuses respondent of unfaithfulness. Husband/partner does not permit respondent to meet female friends. Husband/partner tries to limit respondent’s contact with family. Husband/partner insists on knowing where respondent is.	0 = Never 1 = Often 2 = Sometimes 3 = Yes, but not in the last 12 months	Aggregate responses of ‘never’ to all questions were considered absence of controlling behaviors and coded ‘no.’ Any other responses were recorded as presence of controlling behaviors and coded ‘yes.’
Emotional abuse	Ever been humiliated by husband/partner. Ever been threatened with harm by husband/partner. Ever been insulted or made to feel bad by husband/partner.	0 = Never 1 = Often 2 = Sometimes 3 = Yes, but not in the last 12 months	Aggregate responses of ‘never’ to all questions were considered absence of emotional abuse and coded ‘no.’ Any other responses were recorded as presence of emotional abuse and coded ‘yes.’
Physical violence	Ever been pushed, shook, or had something thrown by husband/partner. Ever been slapped by husband/partner. Ever been punched with fist or hit by something harmful by husband/partner. Ever been kicked or dragged by husband/partner. Ever been strangled or burnt by husband/partner. Ever been attacked with knife/gun or other weapon by husband/partner.	0 = Never 1 = Often 2 = Sometimes 3 = Yes, but not in the last 12 months	Aggregate responses of ‘never’ to all questions were considered absence of physical violence and coded ‘no.’ Any other responses were recorded as presence of physical abuse and coded ‘yes.’
Sexual abuse	Ever been physically forced into unwanted sex by husband/partner. Ever been forced into other unwanted sexual acts by husband/partner. Ever been physically forced to perform sexual acts respondent did not want to.	0 = Never 1 = Often 2 = Sometimes 3 = Yes, but not in the last 12 months	Aggregate responses of ‘never’ to all questions were considered absence of sexual abuse and coded ‘no.’ Any other responses were recorded as presence of sexual abuse and coded ‘yes.’

**Table 2:** Sociodemographic, behavioral, physical, and social environment of the study participants.

Variables	n (%) *	95% Confidence Interval
Wife’s/partnered women’s age		
15–24	737 (24.3)	22.5%, 26.2%
25–34	1144 (37.7)	35.6%, 39.9%
35–49	1153 (38.0)	35.6%, 40.4%
Total	3033	-
Husband’s/partnered men’s age		
17–24	190 (6.3)	5.2%, 7.5%
25–34	1013 (33.4)	31.3%, 35.6%
35–49	1315 (43.4)	41.1%, 45.7%
50+	515 (17.0)	15.3%, 18.8%
Total	3033	-
Residence type		
Urban	949 (31.3)	26.8%, 36.1%
Rural	2084 (68.7)	63.9%, 73.2%
Total	3033	-
Administrative zones		
Western	258 (8.5)	7.2%, 10.1%
Northern	330 (10.9)	9.2%, 12.9%
Central	326 (10.8)	8.9%, 13.0%
Southern Highlands	178 (5.9)	5.0%, 6.8%
Southern	167(5.5)	4.5%, 6.7%
Southwest Highlands	295 (9.7)	8.3%, 11.3%
Lake	888 (29.3)	26.2%, 32.5%
Eastern	501 (16.5)	14.4%, 18.8%
Zanzibar	90 (3)	2.5%, 3.5%
Total	3033	-
Household wealth		
Poor	1133 (37.3)	33.7%, 41.1%
Middle	603 (19.9)	17.9%, 22.0%
Rich	1298 (42.8)	38.7%, 47.0%
Total	3033	-
Wife’s/partnered women’s education		
No formal education	597 (19.7)	17.3%, 22.4%
Primary	1786 (58.9)	56.5%, 61.2%
Secondary+	650 (21.4)	19.5%, 23.5%
Total	3033	-
Husband’s/partnered men’s education		
No formal education	407 (13.5)	11.5%, 15.8%
Primary	1928 (63.9)	61.5%, 66.3%
Secondary+	682 (22.6)	20.5%, 24.9%
Total	3017	-
Wife/partnered women currently working		
No	1138 (37.5)	34.9%, 40.2%
Yes	1895 (62.5)	59.8%, 65.1%
Total	3033	
Husband/partnered men working, last 12 months		
No	290 (9.6)	8.3%, 11.1%
Yes	2735 (90.4)	88.8%, 91.7%
Total	3025	-
Husband/partnered men consume alcohol		
No	2378 (78.4)	76.2%, 80.4%
Yes	655(21.6)	19.6%, 23.8%
Total	3033	-
Wife’s/partnered women’s decision-making autonomy		
High	1442 (48.1)	45.4%, 50.7%
Low	1557 (51.9)	49.3%, 54.6%
Total	2999	-
Wife’s/partnered women’s attitude toward wife-beating		
Favorable	868 (29.9)	27.6%, 32.3%
Unfavorable	238 (70.1)	67.7%, 72.4%
Total	2906	-
Current husband/partnered men expressed any controlling behavior		
No	1233 (40.7)	38.2%, 43.2%
Yes	1800 (59.3)	56.8%, 61.8%
Total	3033	-
Wife/partnered women ever experienced physical abuse		
No	2200 (72.5)	70.4%, 74.6%
Yes	833 (27.5)	25.4%, 29.6%
Total	3033	-
Wife/partnered women ever experienced emotional abuse		
No	2339 (77)	74.9%, 79.2%
Yes	694 (23)	20.8%, 25.1%
Total	3033	-
Wife/partnered women ever experienced sexual abuse		
No	2757 (90.1)	89.4%, 92.2%
Yes	276 (9.1)	7.8%, 10.6%
Total	3033	-

* All numbers are weighted; due to rounding, percentages and total numbers may not add up to 100.

**Table 3:** Adjusted odds ratios (ORs) ** and 95% confidence intervals (95% CIs) for intimate partner violence and women’s attitudes toward wife-beating.

**Variable**	**Any Controlling Behavior**		**Any Emotional Abuse**		**Any Physical Abuse**		**Any Sexual Abuse**	
Attitude toward wife-beating	OR	95% CI	OR	95% CI	OR	95% CI	OR	95% CI
Unfavorable	1		1		1		1	
Favorable	2.04 *	1.63, 2.56	1.88 *	1.45, 2.39	1.98 *	1.55, 2.53	2.12 *	1.47, 3.05

* *p* < 0.05; ** adjusted for age; residence type; administrative zones; household wealth; educational level; working status; alcohol consumption; and women decision-making autonomy.

**Table 4:** Adjusted odds ratios (ORs) and 95% confidence intervals (95% CIs) for intimate partner violence and socio-demographic, behavioral, physical, and social environments.

Variables	Any Controlling Behavior		Any Emotional Abuse		Any Physical Abuse		Any Sexual Abuse	
	OR	95% CI	OR	95% CI	OR	95% CI	OR	95% CI
Wife’s/partnered women’s age								
15–24	1		1		1		1	
23–34	0.99	0.74, 1.34	0.94	0.59, 1.49	0.78	0.55, 1.09	0.50	0.30, 0.84
35–49	1.00	0.70, 1.44	1.01	0.61, 1.66	0.87	0.58, 1.32	0.56	031, 1.04
Husband’s/partnered men’s age								
17–24	1		1		1		1	
25–34	1.10	0.68, 1.78	1.39	0.72, 2.68	1.78 *	1.02, 3.09	1.01	0.42, 2.41
35–49	1.17	0.70, 1.94	1.51	0.74, 3.08	1.68	0.93, 3.06	1.72	0.66, 4.50
50+	0.95	0.54, 1.65	1.77	0.78, 4.03	1.57	0.79, 3.11	1.95	0.70, 5.40
Residence type								
Urban	1		1		1		1	
Rural	0.89	0.65, 1.22	1.05	0.77, 1.43	0.96	0.97, 1.33	0.95	0.57, 1.59
Administrative zones								
Eastern	1	-	1		1		1	
Western	0.95	0.56, 1.61	1.93 *	1.08, 3.48	1.42	0.79, 2.56	2.40 *	1.12, 5.15
Northern	0.84	0.52, 1.35	1.72 *	1.03, 2.88	1.40	0.87, 2.25	2.47 *	1.19, 5.10
Central	0.97	0.58, 1.62	3.34 *	1.91, 5.85	1.50	0.88, 2.55	2.22	0.90, 5.49
Southern Highlands	1.19	0.75, 1.87	1.82 *	1.05, 3.14	1.33	0.84, 2.10	2.06	0.97, 4.38
Southern	0.41 *	0.24, 0.69	1.22	0.61, 2.42	0.41 *	0.18, 0.96	0.13 *	0.02, 0.95
Southwest Highlands	0.44 *	0.28, 0.70	0.87	0.47, 1.61	0.86	0.56, 1.33	1.25	0.56, 2.79
Lake	0.97	0.66, 1.44	3.14 *	1.98, 4.97	1.83 *	1.27, 2.62	2.97 *	1.64, 5.39
Zanzibar	0.85	0.52, 1.39	1.06	0.56, 2.00	0.49 *	0.27, 0.89	0.94	0.39, 2.25
Household Wealth								
Poor	1		1		1		1	
Middle	1.04	0.81, 1.34	0.96	0.71, 1.28	0.81	0.62, 1.05	0.87	0.51, 1.48
Rich	1.36	0.99, 1.88	0.98	0.69, 1.40	0.67 *	0.48, 0.95	1.03	0.60, 1.78
Wife’s/partnered women’s education								
No formal education	1		1		1		1	
Primary	1.68 *	1.28, 2.21	1.35 *	1.01, 1.80	1.22	0.93, 1.61	1.39	0.82, 2.37
Secondary+	2.21 *	1.44, 3.39	1.21	0.72, 2.05	0.97	0.61, 1.56	1.34	0.58, 3.07
Husband’s/partnered men’s education								
No formal education	1		1		1		1	
Primary	0.97	0.71, 1.31	0.98	0.68, 1.39	0.89	0.62, 1.26	0.68	0.44, 1.06
Secondary+	0.97	0.65, 1.43	0.86	0.54, 1.36	0.69	0.44, 1.08	0.80	0.38, 1.71
Wife’s/partnered women currently working								
No	1		1		1		1	
Yes	1.14	0.91, 1.43	1.11	0.87, 1.43	1.22	0.95, 1.57	1.40	0.87, 2.24
Husband/partnered men working, last 12 months								
No	1		1		1		1	
Yes	1.68 *	1.14, 2.48	0.93	0.61, 1.41	1.44	0.97, 2.13	1.29	0.61, 2.70
Husband/partnered men consume alcohol								
No	1		1		1		1	
Yes	1.93 *	1.49, 2.50	2.64 *	2.06, 3.37	2.70 *	2.08, 3.50	2.77 *	1.88, 4.07
Wife’s/partnered women’s decision-making autonomy								
High	1		1		1		1	
Low	1.09	0.89, 1.34	0.91	0.70, 1.19	1.14	0.87, 1.49	1.20	0.81, 1.79

* *p* < 0.05.

## Data Availability

No new data were created or analyzed in this study. Data sharing is not applicable to this article.
